# PHENOTYPES OF CARDIOVASCULAR DAMAGE AND THE RELATIONSHIP WITH COGNITIVE DECLINE: LATENT CLASS ANALYSIS

**DOI:** 10.1093/geroni/igz038.2947

**Published:** 2019-11-08

**Authors:** Lindsay Miller, Chenkai Wu, Calvin Hirsch, Oscar Lopez, Mary Cushman, Michelle Odden

**Affiliations:** 1 Oregon State University, Corvallis, Oregon, United States; 2 Duke Kunshan University, Kunshan, Jiangsu, China; 3 University of California, Davis, Davis, California, United States; 4 University of Pittsburgh, Pittsburgh, Pennsylvania, United States; 5 University of Vermont, Colchester, Vermont, United States; 6 Stanford University, Stanford, California, United States

## Abstract

The association between subclinical cardiovascular disease (SCVD) and cognitive function is well established, however, current tools for addressing subgroups of SCVD have focused on the overall burden of disease. Identifying risky combinations of characteristics may lead to a better understanding of pathophysiologic changes that underlie cognitive decline. Participants included 5,072 older adults from the Cardiovascular Health Study without cardiovascular disease at baseline and followed for 6 years. Using latent class analysis, we identified cardiovascular damage phenotypes based on vascular (internal intima-media thickness, ankle-arm index, white matter grade, and brain infarctions), cardiac (major echocardiogram abnormalities, ST2, and N-terminal pro-brain natriuretic peptide) and inflammatory (interleukin-6, galectin-3, and cystatin C) markers. Using the maximum probability assignment rule, participants were assigned to phenotypes based on the highest posterior probability of membership. We used adjusted linear mixed effects models to evaluate the association between phenotype and cognitive decline, measured annually using the Modified Mini-Mental State Exam. The analysis yielded 5 prevalent phenotypes: healthy (65%), cardiac (10%), inflammatory (10%), multisystem morbidity (11%), and vascular (4%). The vascular phenotype had the greatest rate of decline at 0.88 points per year (95% CI= -1.33, -0.44), followed by the multisystem morbidity phenotype (β=-0.72, 95% CI= -1.07, -0.38), the inflammatory phenotype (β=-0.67, 95% CI= -0.95, -0.38), and the cardiac phenotype (β=-0.45, 95% CI= -0.70, -0.21) compared to the healthy phenotype. Among patterns of cardiovascular damage, vascular damage appears to have the greatest influence on cognitive decline. Identification of these phenotypes may give rise to more informed clinical interventions.

